# Infrared Plasmonic Sensing with Anisotropic Two-Dimensional Material Borophene

**DOI:** 10.3390/nano11051165

**Published:** 2021-04-29

**Authors:** Jingjing Zhang, Zhaojian Zhang, Xiaoxian Song, Haiting Zhang, Junbo Yang

**Affiliations:** 1 Institute of Mirco/Nano Optoelectronic and Terahertz Technology, Jiangsu University, Zhenjiang 212013, China; zhangjingjing13@nudt.edu.cn (J.Z.); songxiaoxian@ujs.edu.cn (X.S.); zhanghaiting@ujs.edu.cn (H.Z.); 2 Department of Physics, College of Liberal Arts and Sciences, National University of Defense Technology, Changsha 410073, China; yangjunbo@nudt.edu.cn; 3 Center of Material Science, National University of Defense Technology, Changsha 410073, China

**Keywords:** plasmonics, sensors, borophene

## Abstract

Borophene, a new member of the two-dimensional material family, has been found to support surface plasmon polaritons in visible and infrared regimes, which can be integrated into various optoelectronic and nanophotonic devices. To further explore the potential plasmonic applications of borophene, we propose an infrared plasmonic sensor based on the borophene ribbon array. The nanostructured borophene can support localized surface plasmon resonances, which can sense the local refractive index of the environment via spectral response. By analytical and numerical calculation, we investigate the influences of geometric as well as material parameters on the sensing performance of the proposed sensor in detail. The results show how to tune and optimize the sensitivity and figure of merit of the proposed structure and reveal that the borophene sensor possesses comparable sensing performance with conventional plasmonic sensors. This work provides the route to design a borophene plasmonic sensor with high performance and can be applied in next-generation point-of-care diagnostic devices.

## 1. Introduction

Surface plasmon polaritons (SPPs), the coherent oscillations of free electrons and photons at the interface of metal and dielectric, have been focused for decades due to the attractive characters such as confining light beyond the diffraction limit and field enhancement on the surface [1,2,3]. Those features have introduced new approaches towards integrated nanophotonics and brought plenty of novel applications such as subwavelength waveguides [4,5], plasmonic metamaterials [6,7], and nanolasers [8,9]. Especially, there is great interest in the application of plasmonic sensing [10,11,12,13,14]. Since SPPs are tightly bound to the metal-dielectric interface, they are very sensitive to the change of local refractive index (RI) of the dielectric, i.e., the environment. Consequently, such change will significantly influence the spectral response of the plasmonic structure, which plays a role in delivering the sensing information [10]. Such property, together with subwavelength footprints, endow plasmonic sensors with high sensitivity, label-free, low cost, and real-time measurement, making it suitable for next-generation point-of-care (PoC) diagnostic devices [15].

During the past decade, two-dimensional (2D) materials have drawn great attention owing to their unique physical and chemical properties [16,17,18]. Because of the high surface-to-volume ratio, ultra-high surface sensitivity, and active tunability, 2D materials also possess great potential in sensing [19,20,21]. In particular, 2D materials such as graphene and black phosphorus (BP) can support deep-subwavelength SPPs in mid-infrared and terahertz (THz) regimes, providing ideal platforms for plasmonic sensing [22,23]. For example, the plasmon resonances of nanostructured graphene can be actively tuned by an external voltage to probe molecules selectively in mid-infrared [24,25]. BP possesses anisotropic plasmons in the plane, which offer richer physics to the sensing design [26,27]. However, low carrier densities (~10^17^ m^−2^) of those 2D materials limit their plasmonic sensing to mid-infrared and THz regimes. Recently, borophene, the 2D boron, has been introduced as a new 2D material, which has great potential in energy and optoelectronic applications [28,29]. Interestingly, borophene is a 2D metal, in spite of the semiconductor nature of its 3D counterpart. Because of the high carrier density (~10^19^ m^−2^), borophene plasmons can reach near-infrared and visible regimes, meanwhile possess in-plane anisotropy [30,31]. Such feature endows borophene with the capabilities of optical modulation, hybridization, and absorption, which can play an important role in nanophotonic devices [32,33,34]. However, the plasmonic sensing of borophene has not been studied yet.

In this work, we propose an infrared plasmonic sensor based on a borophene ribbon array. The ribbons can support localized surface plasmon resonances (LSPRs), which can sense the RI change of the surrounding environment by the spectral shift of the transmission dip. Via analytical and numerical investigation, we analyse the influences of various geometric and material parameters on sensing performance, i.e., the sensitivity and figure of merit, in detail. We show that the sensing performance can be modified and optimized by tuning those parameters, and the proposed borophene plasmonic sensor possesses a comparable sensing level with conventional plasmonic sensors. This work reveals that borophene has great potential in infrared sensing and can be applied in various infrared plasmonic nanodevices.

## 2. Structures, Materials and Methods

**Figure 1:**
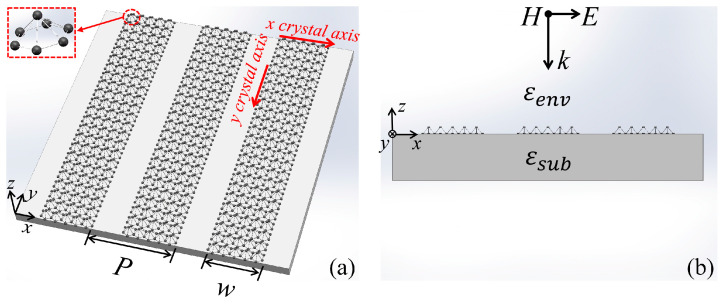
(**a**) The 3D schematic of the proposed plasmonic sensor. It includes a periodic ribbon array with width *w* = 60 nm and period *P* = 200 nm and a substrate with permittivity *ε_sub_* = 1.96. The inset shows the atom structure of borophene. The red arrows indicate the crystal axis of the borophene. (**b**) The front view of the proposed structure. *ε_sub_* represents the permittivity of the substrate, and *ε_env_* represents the permittivity of the environment. *H*, *E*, and *k* indicate the directions of the magnetic field, electric field, and incidence of the plane wave.

The structure of the proposed borophene-based plasmonic sensor is shown in Figure 1a. The borophene is patterned into ribbons with width *w* = 60 nm and period *P* = 200 nm. Such periodic ribbon array is placed on the CaF_2_ substrate with permittivity *ε_sub_* = 1.96 [35]. The in-plane anisotropic conductivity of borophene is described by the Drude model [31]:

(1) $$\sigma_{jj}(\omega )=\frac{iD_{j}}{\pi (\omega +i\tau^{-1})},\;D_{j}=\frac{\pi e^{2}n_{s}}{m_{j}}$$

where *j* indicates the direction of optical axes of borophene crystal and can be chosen as *x* or *y*. *ω* is the angular frequency of light, and *τ* is the electron relaxation time and initially set as 65 fs. *D_j_* represents the Drude weight, where *e* is the electron charge, *n_s_* is the electron density, and *m_j_* is the effective electron mass in two crystal axis directions. Here, *α* phase borophene, which possesses strong anisotropy as well as high electron density, is chosen in this study [31]. Thus, *n_s_* is initially set as 5 × 10^19^ m^−2^, *m_x_* = 1.4 *m*_0_, and *m_y_* = 5.2 *m*_0_, where *m*_0_ represents the rest mass of the electron. Those geometric and material parameters remain unchanged unless otherwise stated.

Finite-Difference Time-Domain (FDTD) method (performed in Lumerical FDTD solution) is employed to implement the full-wave numerical calculation. Since the structure is homogeneous along ± *y* directions, the 2D numerical calculation is performed in the *x*-*z* domain of the structure, which treats the structure as infinite along ± *y* directions. In the computational domain, the unit cell of the structure is established with periodic boundary conditions in ± *x* directions, and perfectly matched layers (PMLs) with 8 layers are set along ± *z* directions. Fine meshes with *dx* = 0.5 nm and *dz* = 0.1 nm are used for the discretization to ensure the convergence of the numerical results. A plane wave source with *x*-polarization is illuminated from the top of the structure to perform the far-field excitation as presented in Figure 1b, and a planar power monitor is placed at the bottom of the structure to detect the total transmitted power *P_t_*. The normalized transmission as a function of the wavelength is calculated via *T*(*λ*) = *P_t_*(*λ*)/*P_s_*(*λ*), where *P_s_* is the total power of the plane wave source.

## 3. Theory of Proposed Plasmonic Sensing

The proposed ribbon array can support localized resonant SPPs, namely, LSPRs, which originate from the Fabry–Perot (F–P) resonances on the surfaces of ribbons [36]. Neglecting the interaction within adjacent unit cells, the resonant condition of LSPR in one ribbon is as follows [37]:

(2) 2Re(ksp)w+2ϕ=2mπ, m=0,1,2…

where *k_sp_* is the wave vector of SPPs on the ribbon; *ϕ* is the phase shift at the edges of ribbon due to the reflection; *m* is the resonant order, which is the integer number. Since *k_sp_* = *n_eff_ k_LSPR_* = *2πn_eff_*/*λ_LSPR_*, where *n_eff_* is the effective RI of SPPs, *k_LSPR_* is the resonant wave vector in free space, and *λ_LSPR_* is the resonant wavelength, Equation (2) can be rewritten as follows:

(3) $$2wn_{eff}=(m-\frac{\phi }{\pi })\lambda_{LSPR}=n\lambda_{LSPR}$$

where *n* is the non-integer number indicating the resonant order. Considering that borophene is a 2D conductor without thickness, it can be regarded as a conductive boundary condition when solving Maxwell’s equations; thus, the dispersion equation of SPPs on the borophene, within the quasistatic limit, can be described as follows [38]:

(4) $$k_{sp}=\frac{i(\varepsilon_{env}+\varepsilon_{sub})\varepsilon_{0}c}{\sigma_{jj}(\omega )}k_{0}$$

where *ε*_0_ is the permittivity of vacuum; *c* is the velocity of light in vacuum; *k*_0_ is the wave vector in free space and, here, *k*_0_ = *k_LSPR_*. Combining Equations (1), (3), and (4), we can get an impression of resonant wavelength as follows:

(5) $$\lambda_{LSPR}=2\pi c\sqrt{\frac{\varepsilon_{0}w(\varepsilon_{env}+\varepsilon_{sub})}{nD_{j}}}$$

The sensory sensitivity is defined as the resonant wavelength shift per unit RI change of the environment [39]:

(6) $$S=\frac{\partial \lambda_{LSPR}}{\partial n_{env}},\;n_{env}=\sqrt{\varepsilon_{env}}$$

where *n_env_* is the RI of the environment and initially set as 1. We can derive the sensitivity from Equations (5) and (6) as follows:

(7) $$S=2\pi c\sqrt{\frac{\varepsilon_{0}w}{nD_{j}}}\frac{n_{env}}{\sqrt{n_{env}^{2}+\varepsilon_{sub}}}=\frac{n_{env}}{n_{env}^{2}+\varepsilon_{sub}}\lambda_{LSPR}$$

Thus, the influences of material and geometrical parameters on the sensitivity can be analytically acknowledged from Equation (7). Besides, another important sensing factor is the figure of merit (FOM), which indicates the optical resolution of the sensing [39]:

(8) $$\mathrm{FOM}=\frac{S}{\mathrm{FWHM}}$$

where FWHM is the full width of half maximum of the resonance. FWHM is decided by the internal decay rate *τ*^−1^ due to the material intrinsic loss, as well as external decay rate $\tau_{ext}^{-1}$ due to the leakage radiative loss:

(9) $$\mathrm{FWHM}=2\tau_{tot}^{-1}=2(\tau^{-1}+\tau_{ext}^{-1})$$

where $\tau_{tot}^{-1}$ is the total decay rate. Since the external decay rate cannot be analytically described, FWHM can be calculated by fitting the numerically calculated transmission spectrum with the Fano formula [40]:

(10) $$T_{Fano}={\left|a_{1}+ia_{2}+\frac{b}{\lambda -\lambda_{LSPR}+i\tau_{tot}^{-1}}\right|}^{2}$$

where *a*_1_, *a*_2_, and *b* are fitting real fitting parameters.

## 4. Simulation and Discussion

**Figure 2:**
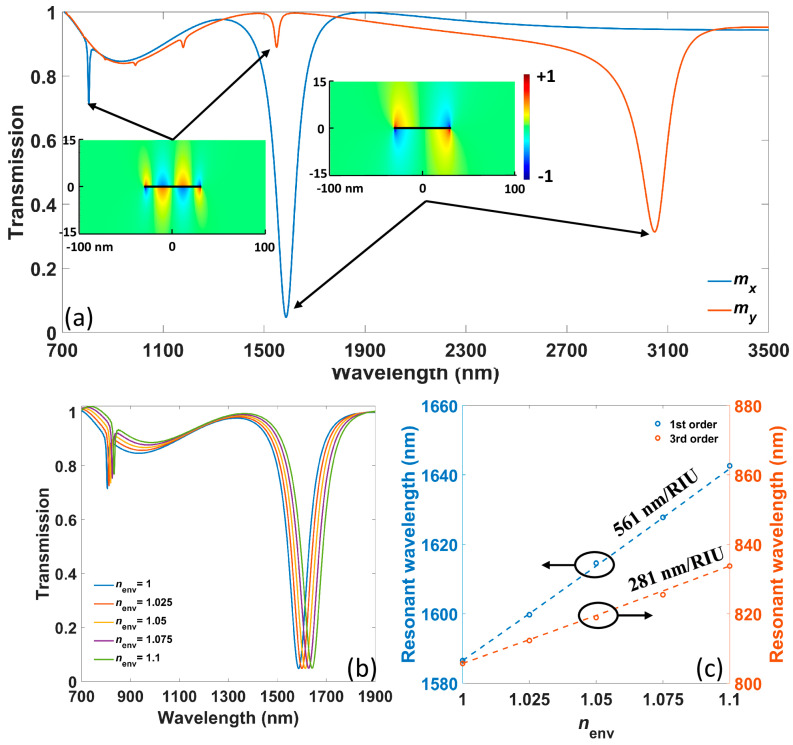
(**a**) The numerically calculated transmission spectra of proposed ribbon array when the *x*-axis and *y*-axis of borophene are arranged along the *x*-direction of the coordinate system, respectively. The insets show *E_z_* distributions of resonances, and the black lines within the field indicate the position of the borophene ribbon. (**b**) The transmission spectra under different *n_env_* from 1 to 1.1 with the step 0.025. (**c**) The relation between resonant wavelengths and *n_env_* for the two resonances. The circles are numerical results, and dotted lines indicate analytical results.

The numerically calculated transmission spectra of the proposed ribbon array are shown in Figure 2a. The blue and orange curves are spectra when the *x* (as marked in the inset of Figure 1a) and *y* crystal axes of borophene are arranged along the *x*-direction of the coordinate system, respectively. For the first case, it is shown that there are two resonant dips at 806 nm and 1586 nm in the near-infrared regime, respectively. The insets present the normalized distributions of the *z* component of the electric field (*E_z_*) for each resonance. The fields possess typical F–P mode profiles, and there are three nodal lines for the mode at 806 nm and one for the mode at 1586 nm. Thus, the first mode is corresponding to the third-order F–P resonance when *m* = 3 and the second one is the first-order F–P resonance with *m* = 1. It has to be mentioned that the fundamental (*m* = 0) and second-order (*m* = 2) F–P resonances cannot be excited by the plane wave due to the parity mismatch of electromagnetic fields. When the *y*-axis of borophene is arranged along the *x*-direction, the third-order F–P resonance red-shifts to 1550 nm, and the first-order F–P resonance red-shifts to 3047 nm coming to the mid-infrared regime. This is due to the larger electron mass of borophene along the *y*-axis, which leads to lower Drude weight as well as longer resonant wavelengths, according to Equation (5). We will focus on the case of the *x*-axis of borophene along the *x*-direction in the following contents unless otherwise stated.

When the RI of environment *n_env_* is changed (which can be induced by the change of solution concentration, DNA hybridization process and so on), the resonance, according to Equation (5), will redshift to sense such change. The numerically calculated transmission spectra under different *n_env_* from 1 to 1.1 with the step 0.025 are shown in Figure 2b, and the corresponding resonant wavelengths are plotted with circles in Figure 2c. The numerical results can also be fitted by analytical results calculated from Equation (5), where the reflected phase shift *ϕ* is 0.3 *π*, which is consistent with previous experimental results [37]. The analytical results are marked by dotted lines in Figure 2c, which agree with numerical results. It is indicated that there is an approximately linear relation between resonant wavelengths and *n_env_*, and the sensitivity is 561 nm shift per refractive index units (561 nm/RIU) for the first-order resonance and 281 nm/RIU for the third-order resonance. Such sensitivity difference can be explained by Equation (7), indicating that higher-order resonances possess shorter resonant wavelengths that lead to lower sensitivity. Since the resonant intensity of third-order resonance is weak and can hardly be detected, we will focus on first-order resonance in the following contents.

**Figure 3:**
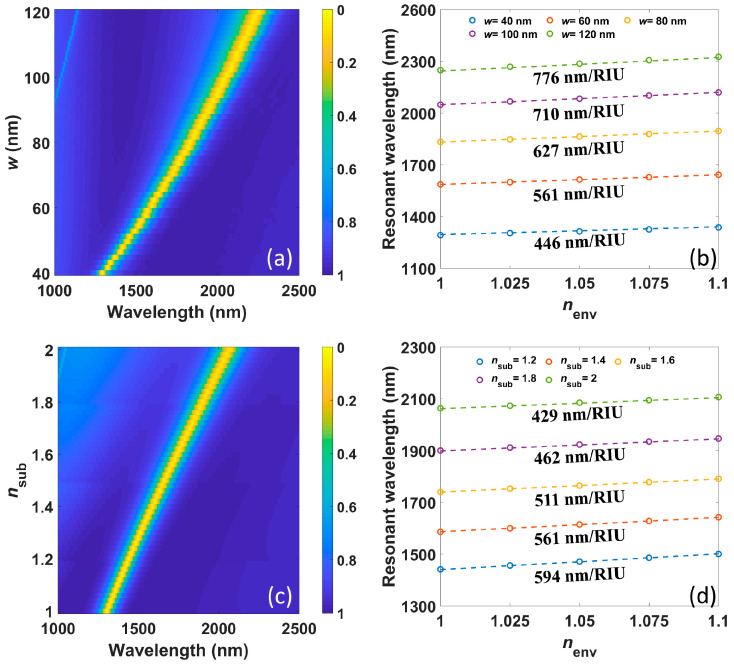
(**a**) The numerically calculated transmission spectra under different ribbon widths *w*; meanwhile, the ratio *w*/*P* remains at 0.3. (**b**) The numerically (circles) as well as analytically calculated (dotted lines) resonant wavelengths versus *n_env_* under different *w*. (**c**) The numerical transmission spectra under different RI of the substrate *n_sub_*. (**d**) The numerically (circles) as well as analytically calculated (dotted lines) resonant wavelengths versus *n_env_* under different *n_env_*.

Figure 3a presents numerically calculated transmission spectra under different ribbon width *w*; meanwhile, the ratio *w*/*P* remains at 0.3 to keep the intercellular interaction constant. It is shown that the resonant wavelengths of first-order resonance redshift with increasing *w*, which is consistent with Equation (5). The numerically as well as analytically calculated resonant wavelengths versus *n_env_* under different *w* are provided in Figure 3b, which show that the sensitivity increases from 446 nm/RIU to 776 nm/RIU when *w* increases from 40 nm to 120 nm. Thus, Equation (7) successfully predicts the evolution of sensitivity. Figure 3a also indicates that FWHM of the resonance becomes wider with increasing *w*, which can be ascribed to higher material loss at a longer wavelength, as well as the higher radiative loss due to the expansion of the resonant area, according to Equation (9). Consequently, the corresponding FOM decreases from 6.75 to 4.39/RIU. There is a trade-off between sensitivity and FOM here.

The numerically calculated transmission spectra versus different RI of substrate *n_sub_* are depicted in Figure 3c. Expectantly, the resonant wavelengths red-shift with increasing *n_sub_* that agrees with Equation (5). The numerically and analytically calculated resonant wavelengths versus *n_env_* under different *n_sub_* are in good agreement, as shown in Figure 3d, and the sensitivity declines from 594 nm/RIU to 429 nm/RIU as *n_sub_* increases from 1.2 to 2. Such declining tread of sensitivity is also well indicated in Equation (7). With a longer resonant wavelength, The FWHM of the resonance increase much less in Figure 3c compared with that in Figure 3a because the radiative loss is almost unaffected by *n_sub_*, and only material loss contributes to the increase of total loss. The corresponding FOMs are 7.20, 6.07, 4.92, 3.94 and 3.25/RIU, respectively, which also drop continuously.

**Figure 4:**
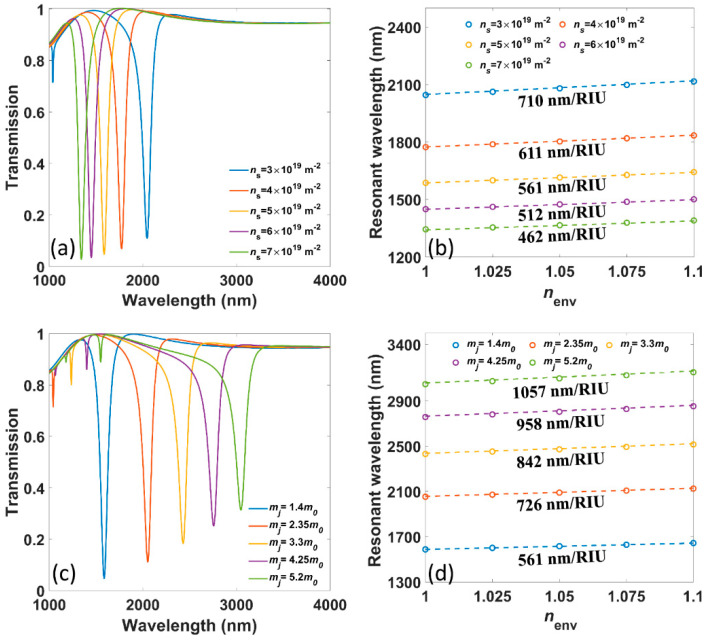
(**a**) The numerically calculated transmission spectra under different electron densities *n_s_*. (**b**) The numerically (circles) as well as analytically calculated (dotted lines) resonant wavelengths versus *n_env_* under different *n_s_*. (**c**) The numerically calculated transmission spectra under different electron masses *m_j_*. (**d**) The numerically (circles) as well as analytically calculated (dotted lines) resonant wavelengths versus *n_env_* under different *m_j_*.

Similar to graphene and BP, the electron density of borophene can be actively tuned by chemical doping or external voltage. The numerically calculated transmission spectra under different electron density *n_s_*, from 3 × 10^19^ m^−2^ to 7 × 10^19^ m^−2^, are plotted in Figure 4a. The blueshift of resonant wavelength happens when *n_s_* increases, which is attributed to higher Drude weight according to Equation (5). Besides, under higher *n_s_*, the resonant intensity becomes stronger, and the FWHM gets narrower, which is attributed to both less material loss in a shorter wavelength range and more charge carriers that get coupled in the oscillation. In Figure 4b, the analytical and numerical results of resonant wavelengths versus *n_env_* with different *n_s_* show that the corresponding sensitivity decreases from 710 nm/RIU to 462 nm/RIU, which is consistent with the prediction of Equation (7). At the same time, the FOM decrease from 6.83 to 5.09/RIU, showing a positive correlation with sensitivity. Such dynamic tunability of the proposed sensor makes it possible to selectively sense the wavelength regime of interest. 

The electron mass *m_j_* along different crystal axes of borophene affects the behaviors of SPPs significantly, which leads to anisotropic plasmons in the borophene surface. To reveal the role of *m_j_* in plasmonic sensing, *m_j_* is artificially increased from 1.4 *m*_0_, the value of *m_x_*, to 5.2 *m*_0_, which is the value of *m_y_*. The corresponding numerically calculated transmission spectra are presented in Figure 4c, showing that the resonant wavelength, as well as FWHM, increase with larger *m_j_*, which is due to lower Drude weight according to Equation (5) and higher intrinsic loss, respectively. The numerical and analytical results in Figure 4d show that the sensitivity raises from 561 nm/RIU to 1057 nm/RIU, which can also be well predicted by Equation (7). The corresponding FOMs are 6.07, 6.87, 7.29, 7.53 and 7.44/RIU, respectively. Notably, there is a fluctuation in the evolution of FOM in this case, where FOM rises first and then falls.

**Figure 5:**
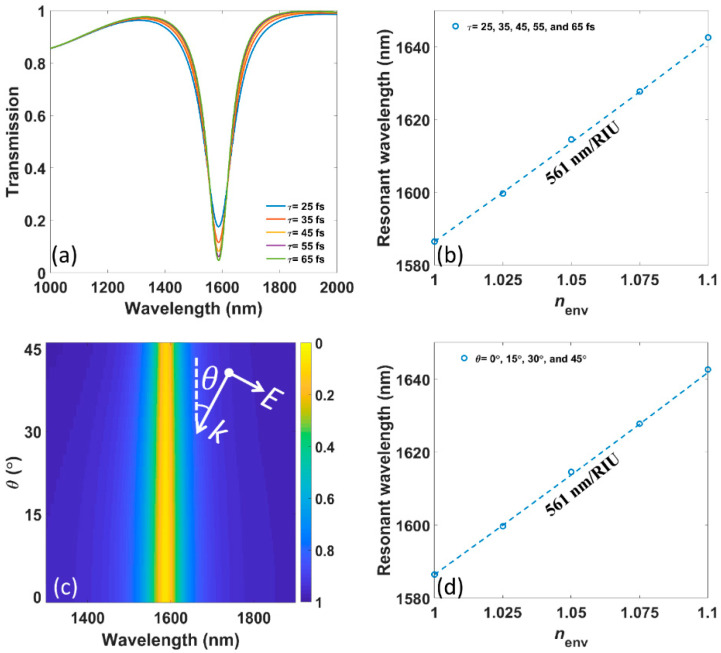
(**a**) The numerically calculated transmission spectra under different electron relaxation times *τ*. (**b**) The numerically (circles) as well as analytically calculated (dotted lines) resonant wavelengths versus *n_env_* under different *τ*. (**c**) The numerically calculated transmission spectra under different incident angles *θ*. The inset shows the definition of *θ*. (**d**) The numerically (circles) as well as analytically calculated (dotted lines) resonant wavelengths versus *n_env_* under different *θ*.

Moreover, transmission spectra and sensing performances under different electron relaxation times *τ* are illustrated in Figure 5a,b, respectively. It is shown that *τ* does not influence the resonant wavelength but only enhances the resonance and narrows the FWHM as it becomes longer. The sensitivity remains at 561 nm/RIU as shown in Figure 5b, and FOM continuously rises from 4.47 to 6.07/RIU. Those phenomena are consistent with the theory since *τ* is not shown in Equations (5) and (7) but only gets involved in Equation (9). The reciprocal relation indicates that a longer relaxation time brings lower internal decay rate, that is, less intrinsic loss.

We also investigate the influence of incident angle *θ*, which is marked in the inset of Figure 5c, on the resonant wavelength as well as sensing performance, which is presented in Figure 5c,d, respectively. Here, Bloch boundary conditions are used in ±*x* directions to ensure the correction of the numerical results. It is shown that both resonant wavelength and sensitivity do not depend on the incident angles from 0° to 45°. From Equation (7), we can conclude that the invariance of sensitivity is inherited from the unchanging resonant wavelength. Due to the structural symmetry, the same conclusion holds for angle from −45° to 0°. Actually, such a phenomenon is common in LSPRs based on other 2D materials such as graphene, and the invariances can also be expected for a larger angle of incidence [41,42]. This is because LSPRs in 2D materials are in deep-subwavelength and ultra-confined. Meanwhile, as shown in Figure 5c, the FWHM becomes a little narrower with increasing *θ*, leading to the increasing FOM from 6.07 to 9.01/RIU. That indicates the oblique incident angle can reduce the radiative loss of the resonance.

**Figure 6:**
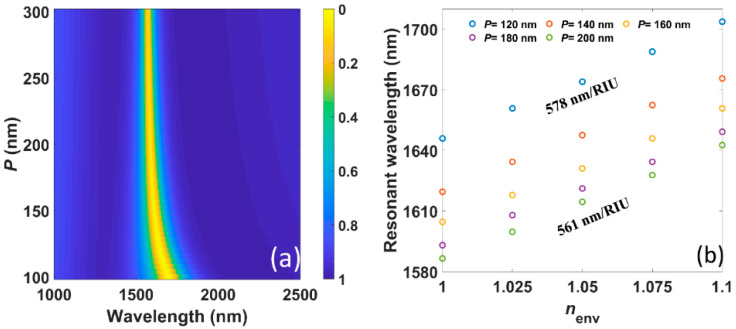
(**a**) The numerically calculated transmission spectra under different period *P* while *w* is kept as 60 nm. (**b**) The numerically calculated (circles) resonant wavelengths versus *n_env_* under different *P*.

Furthermore, we numerically study the influence of the intercellular coupling, i.e., the period *P*, on the resonant wavelength as well as sensing performance, which is not involved in theory in Section 3. The numerically calculated transmission spectra under different *P* (*w* is kept as 60 nm) in Figure 6a show that there is a blueshift with increasing *P*, consistent with previous studies [43,44]. Meanwhile, the FWHM goes much narrower with larger *P* owing to the decrease of both intrinsic and radiative loss, which indicates that the strong interaction of resonances in adjacent unit cells will boost the radiative loss of the system. The sensitivity, which is given in Figure 6b, is 578 nm/RIU when *P* = 120 nm, and then remains at 561 nm/RIU from *P* = 140 nm to 200 nm. It is due to the invariant resonant wavelength with a sufficiently large *P*. At the same time, the FOM continuously rises from 3.80 to 6.07/RIU.

Now, we will discuss how to enhance the sensitivity meanwhile maintain a high FOM. Analytical and numerical results confirm that longer resonant wavelength can lead to higher sensitivity, and the corresponding approaches to increase the resonant wavelength include increasing ribbon width *w* and electron mass *m_j_*, as well as decreasing electron density *n_s_* and period *P*. Notably, the only exception is RI of substrate *n_sub_*, which influences both resonant wavelength and sensitivity directly. The investigation above indicates that lower *n_sub_* benefits the sensitivity even it reduces the resonant wavelength. However, there is a trade-off between sensitivity and FOM when changing *w*, *m_j_* and *P*, so one should consider the balance between sensitivity and FOM when changing those parameters. There is a positive correlation between sensitivity and FOM when tuning *n_sub_* and *n_s_*; thus, those two parameters are ideal for boosting both sensitivity and FOM. The electron relaxation time *τ* and incident angle *θ* cannot influence the resonant wavelength as well as sensitivity, but help improve FOM when they are in larger values.

Finally, we will compare borophene plasmonic sensors with conventional plasmonic sensors based on metal and graphene. Since the resonant wavelength matters for the sensitivity, we will use the normalized sensitivity *S*’ = *S*/*λ_LSPR_* to compare sensitivity fairly. For metal particle arrays in the near-infrared region, the *S’* is approximately 0.59, and the corresponding FOM is approximately 1.1 [39]. For graphene ribbon arrays in the mid-infrared region, the *S’* is approximately 0.29, and the corresponding FOM is approximately 5 [45]. The average *S’* and FOM in this work are 0.35 and 5.5, respectively, showing comparable levels of sensing performances. Notably, compared with metal sensors, borophene sensors possess a much smaller footprint as well as electrical tunability. Compared with graphene sensors, the sensing spectral regime of borophene sensors is richer, ranging from visible, near-infrared, to mid-infrared light.

## 5. Conclusions

In conclusion, we proposed an infrared plasmonic sensor based on nanostructured borophene. Utilizing LSPRs supported on the borophene ribbon array, a resonant dip can be generated in the transmission spectrum, which can sense the local RI information of the surrounding environment. The analytical and numerical results are in good agreement. Additionally, we investigated the influences of various geometric and material parameters on the sensing performances as well as the corresponding physical mechanisms in detail. Finally, we discussed the schemes to enhance the sensing performance and make comparisons. This work reveals the potential applications of borophene plasmon in next-generation PoC diagnostic systems and can provide useful information for the design of various borophene-based optoelectronic and photonic nanodevices.

## Data Availability

The data presented in this study are available on request from the corresponding author. The data are not publicly available due to personal data protection.
